# Did the genus *Parandrocephalus* Heller, 1916 (Coleoptera, Cerambycidae, Callichromatini) cross the Wallace line? The taxonomic status of *Parandrocephalus blairi* Bentanachs & Vives 2009 and a new subgenus of *Hexamitodera* Heller, 1896, with notes on convergent evolution and secondary sexual characters

**DOI:** 10.3897/zookeys.293.5133

**Published:** 2013-04-19

**Authors:** Robert Perger

**Affiliations:** 1 Colección Boliviana de Fauna. Casilla 8706, La Paz, Bolivia

**Keywords:** Longhorn beetles, Malaysia, Indonesia, Sulawesi, Wallacea, taxonomy, biogeography

## Abstract

The genera *Parandrocephalus* Heller, 1916 and *Hexamitodera* Heller, 1896 are reviewed and redescribed. Based on the combination of chromatic sexual dimorphism, velvety pubescence on the whole dorsal body and distinctly developed carina on the elytra, *Parandrocephalus blairi* Bentanachs & Vives, 2009 is transferred to *Hexamitodera*.

A new subgenus, *Sulcognatha* Perger, is instituted to accommodate mandible, head and metasternal modifications in *Hexamitodera blairi* **comb. n.** that are lacking in the type species of *Hexamitodera*, *Hexamitodera semivelutina*. As indicated by fundamental structural differences in the mandibles of *Parandrocephalus* and *Hexamitodera (Sulcognatha) blairi* **comb. n.**, the exaggerated secondary sexual traits and open procoxal cavities in both taxa are presumably the result of convergent evolution. Contrary to Bentanachs & Vives (2009), the presence of the two *Parandrocephalus* species in Sundaland and the endemism of *Hexamitodera* on Sulawesi agree well with the zoogeographical separation of both areas by the Wallace line.

## Introduction

The discovery and description of two distinct zoogeographical realms in South East Asia by Alfred Russel Wallace was the birth of biogeography and inspired the naturalist to formulate fundamental principles of the evolution theory, at the same time with Charles Darwin (Sarkar 1998). Like Darwin, Wallace was genuinely interested in beetles, however, both never pursued beetle taxonomy (see Beutel et al. 2009), nor did Wallace compare the beetle faunas of the zoogeographical realms he discovered, likely because of the enormous and difficult manageable diversity of this taxon.

A beetle group that has attracted the attention of following generations of collectors and taxonomists and is mainly speciose in the tropical regions of the Austral hemisphere is the heterogeneous tribe Callichromatini (Schmidt 1922).

Taxa with a combination of chromatic sexual dimorphism and velvety pubescence on the whole dorsal body are exceptional for this tribe. Only three Asian Callichromatini species share this combination: *Hexamitodera semivelutina* Heller, 1896, *Parandrocephalus blairi* Bentanachs & Vives, 2009 and *Niisatochroma celebiana* Vives & Bentanachs, 2010, all occurring on Sulawesi.

Particularly *Parandrocephalus blairi* with the con-generics *Parandrocephalus eversor* Heller, 1916 and *Parandrocephalus drescheri* Blair, 1938 on the Sunda Islands represents an interesting case as it belongs to one of the few Cerambycidae genera that occur on both sides of the Wallace line (Bentanachs and Vives 2009).

In this study I review generic characters to test the hypothesis of whether the genus *Parandrocephalus* indeed crossed the Wallace line or alternatively, the Sulawesian taxa share characters that rather indicate vicariance processes and support the Wallace line as an effective zoogeographical boundary.

### Materials and methods

Specimens examined for this study are from the following institutions / private collections**:**

**BMNH** Natural History Museum, London, U.K.;

**JA** Dr. Joachim Adolphi, Private collection, Dresden, Germany;

**RMNH **National Museum of Natural History in Leiden, The Netherlands;

**RP** Robert Perger, Private collection, Santa Cruz, Bolivia;

**RV **Robert Vigneault, Private collection, Quebec, Canada;

**SNSD**Senckenberg Naturhistorische Sammlungen Dresden,Germany;

**TN** Tatsuya Niisato, Private collection, Tokyo, Japan;

**UN** Ulf Nylander, Private collection, Valbo, Sweden.

Morphological characters were examined with a stereomicroscope and specimens were photographed with a Canon 450D reflex camera fitted with macro lenses.

The following specimens were examined:

***Parandrocephalus eversor* Heller, 1916**

Sumatra, Padangsche Bovenlanden: holotype 1 ♂, RMNH, ex coll. Dr. H. J. Veth; Malaysia, Cameron Highlands, Kampong Rajah: 1 ♂, RP, VI-2000; Borneo, Sabah, Mt. Trus Madi: 1 ♀, UN, 19-VI-05, S.Chew coll.

***Parandrocephalus drescheri* Blair, 1938**

Java, G. Tangkoeban Prahoe, 4000-5000 voet [= ft.]: holotype,1♂, BMNH,VI-1937, F.C. Drescher coll.[G. Tangkoeban Prahoe, 4000-5000 voet [= ft.], Dreanger, Java, VI.1937 / type / Parandroceph. drescheri Blr., ♂ type det. K.G. Blair / Brit.Mus., 1937-662]

***Parandrocephalus blairi* Bentanachs & Vives, 2009**

Indonesia, Sulawesi, Puncak near Palolo: holotype, 1 ♂, TN, II-1990; Indonesia, Sulawesi, Palolo Palu: allotype, 1 ♀, TN, IV-1991; Indonesia, Central Sulawesi, Palolo Palu: 2 ♂ RP, 1 ♂ JA, III-1999; Indonesia, Central Sulawesi, Puncak: 1 ♂, RP, IV-1999.

***Hexamitodera semivelutina* Heller, 1896**

Indonesia, North Sulawesi: holotype, 1 ♀, SNSD [semivelutina Hh. / N.Celebes, 9389 / Typus / Staatl. Museum für Tierkunde, Dresden]; Indonesia, Sulawesi, Minado: 1 ♂, BMNH, 1781, Wallace coll., ex coll. Fry; Indonesia, Sulawesi: 1 ♀, BMNH, 1922; Indonesia, Sulawesi, Pulu Pulu: 1♂, RV, 19-XII-1997, A. Audureau coll.

## Results

### Parandrocephalus

Heller, 1916

http://species-id.net/wiki/Parandrocephalus

#### Type species.

*Parandrocephalus eversor* Heller, 1916

#### Redescription.

Body relatively large, elongated, parallel-sided, flattened, without chromatic sexual dimorphism. Head and mandibles with pronounced sexual dimorphism, abnormally enlarged in male, vertex glabrous. Male mandible sickle-shaped, flattened vertically, with dorsomedian carina, median longitudinal concave. Antennae slightly surpassing the basal elytral half (not the apical third of the elytra as diagnosed by Bentanachs and Vives 2009). Pronotum transverse, glabrous, obtusely toothed at each side, deeply constricted anteriorly and posteriorly; anterior margin strongly projected forward. Procoxal cavities open posteriorly. Elytra covering the abdomen, sparsely pubescent, with scarcely elevated costae. Female abdominal sternites1**–**4 distally margined with whitish pubescence. Metatibia apically distinctly flattened and broadened, about as wide as metafemora.

### Parandrocephalus eversor

Heller, 1916

http://species-id.net/wiki/Parandrocephalus_eversor

Fig. 1A, B

#### Redescription.

Male. Body 4.2 times as long as wide. Head and pronotum (Fig. 2B) glabrous. Head dorsally polished, coarsely wrinkled, vertex strongly convex, not carinated dorsally; frons strongly concave, with a deep longitudinal furrow. Temple in dorsal view strongly convex; distance between temples wider than anterior border of pronotum and as wide as widest width of pronotum. Gena apically rounded, externally thickened. Width between genae as wide as width of head. Mandible sickle-shaped, curved, forming an elongated ellipse in closed position, with dorsomedian carina; apex conical, unidentate; molar margin without basal tooth. Antenna sparsely pubescent, bicolored. Elytra 2.2 times as long as prothorax and head excluding mandibles.

Female.Vertex and temples straight, distance between temples narrower than anterior pronotal margin and widest pronotal width, vertex setose, with longitudinal furrow reaching anterior pronotal margin. Mandible horizontally flattened, dorsal surface convex, with dorso-lateral carina, externally straight. Pronotal disc setose. Elytra 3.43 times as long as prothorax and head excluding mandibles. Abdomen bordered with yellowish pubescence at the base of all ventrites.

#### Geographical distribution.

Sumatra, Borneo, Malaysia Peninsula.

**Figure 1.**
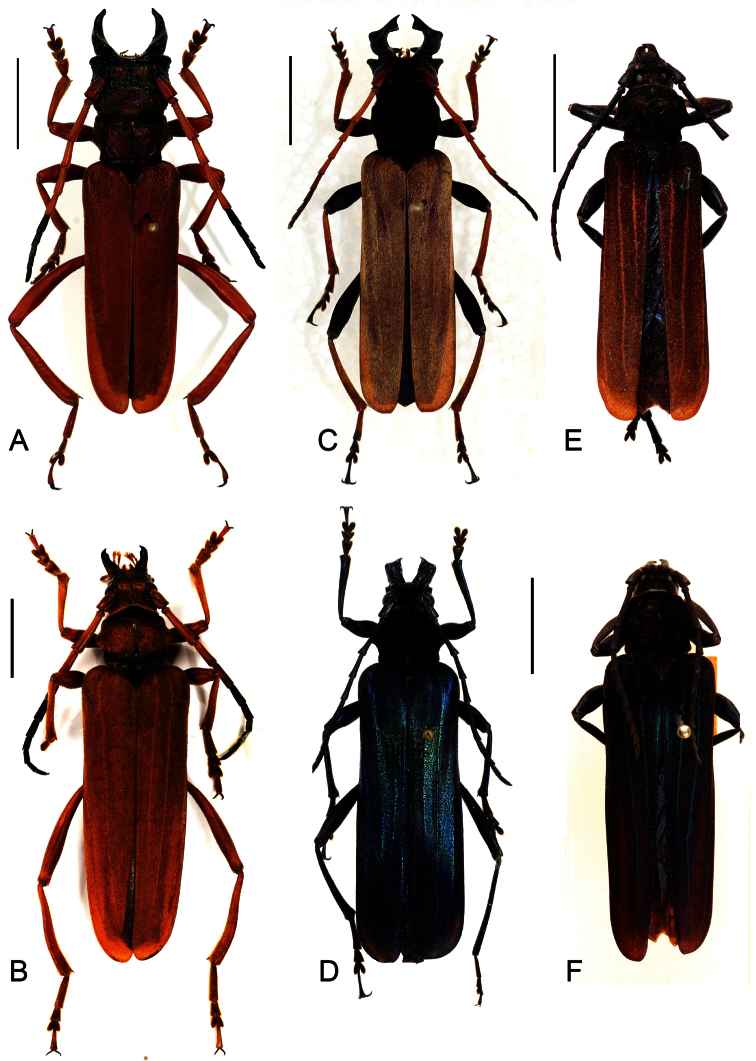
Dorsal view habitus, scale bars 10 mm **A** *Parandrocephalus eversor*, male, Malaysia, Cameron Highlands, Kampong Rajah **B** idem, female **C** *Hexamitodera (Sulcognatha) blairi* comb. n., male, Indonesia, Central Sulawesi, Puncak **D** idem, female, allotype, Indonesia, Sulawesi, Palolo Palu **E** *Hexamitodera semivelutina*, male, Sulawesi **F**, idem, female, holotype, Indonesia, North Sulawesi.

**Figure 2.**
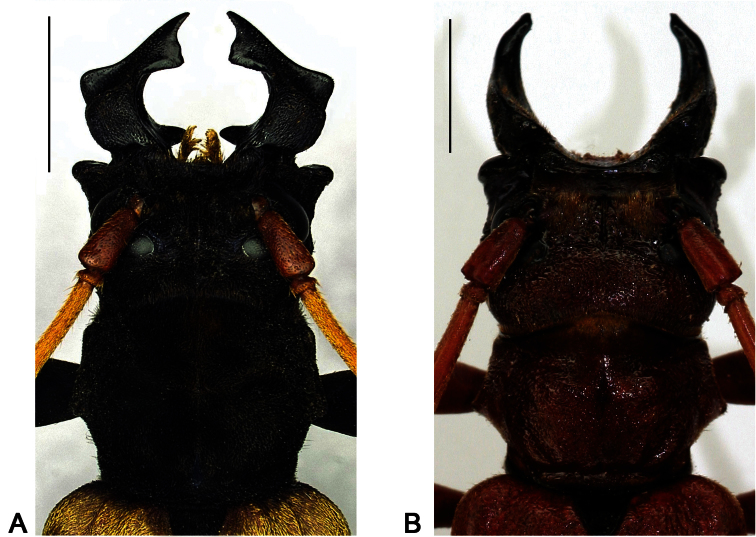
Dorsal view head and pronotum, scale bars 5 mm **A** *Hexamitodera (Sulcognatha) blairi* comb. n., male, Indonesia, Central Sulawesi, Puncak **B** *Parandrocephalus eversor*, male, Malaysia, Cameron Highlands, Kampong Rajah.

### Hexamitodera

Heller, 1896

http://species-id.net/wiki/Hexamitodera

#### Type species.

*Hexamitodera semivelutina* Heller, 1896 (monotypic)

#### Redescription.

Body relatively large, elongated, flattened, dorsally covered with dense velvety pubescence (could be partially abrased, particularly in females), with chromatic sexual dimorphism. Mandible horizontally flattened, dorsal surface straight, without dorsomedian carina, dorso-lateral border, externally straight or with shallow to deep concavity. Antenna relatively short, reaching or slightly projecting above basal half of elytra. Pronotum transverse, obtusely to acutely toothed laterally; apical margin not constricted. Procoxal cavity open posteriorly. Sternum and epimeron of meso- and metathorax densely pubescent. Elytra covering abdomen, parallel-sided, with three distinctly elevated longitudinal costae, the inner two converging in about the apical third of the elytra. First abdominal sternite of female distally bordered with white pubescence. Femur fusiform, comparably short, stout; metatibia moderately flattened and widened, narrower than metafemur.

### Hexamitodera semivelutina

Heller, 1896

http://species-id.net/wiki/Hexamitodera_semivelutina

Fig. 1E, F

#### Redescription.

**Figure 3.**
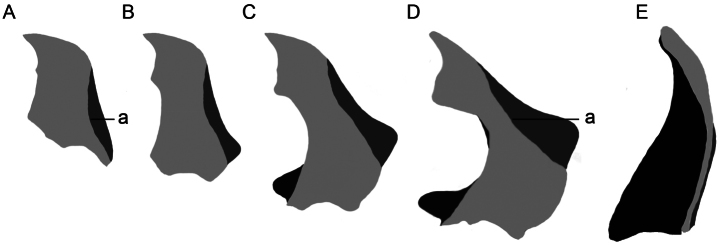
Dorsal view male mandibles; light grey, dorsal surface; dark grey, ventral external concavity; black, interior surface; a, dorso-lateral border; mandibles of **A** and **D** are drawn from specimens that were digitally scaled until they had similar body length **A** *Hexamitodera semivelutina* **B, C** hypothetical intermediate forms **D** *Hexamitodera (Sulcognatha) blairi* comb. n. **E** *Parandrocephalus eversor*.

Male. Body 3.7–3.8 times as long as wide. Vertex and temples straight, distance between temples narrower than anterior margin of pronotum and widest pronotal width. Head dorsally uniformly finely punctured, with a fine longitudinal furrow reaching the base. Mandible without inner basal tooth, feebly curved or angulate (Fig. 3A). Mandible as long as one-half of the rest of the head, as long and wide as scape. Gena rounded apically; width between genae distinctly narrower than width between temples, the latter narrower than pronotal width. Antenna sparsely pubescent. Antennomeres 1**–**2 apically rounded, 3 straight, as 1.9 times as long as scape, 4**–**11 obtusely toothed. Antenna and legs unicolorous.

Prothorax 1.21 times as wide as long. Procoxal cavity opened posteriorly by a comparable small gap. Apex of mesosternal process not concealed by metasternum.

Elytra 2.9 times as long as prothorax and head excluding mandible and 2.8 times as long as elytra width, brownish.

Female. Head, mandible and pronotum as in male. Elytron 3.3 times as long as prothorax and head excluding mandible, metallic blue with purple brownish stripes and brown apices.

#### Geographical distribution.

Sulawesi.

### Sulcognatha

Perger subgen. n.

Fig. 1C, D

#### Type species.

*Hexamitodera (Sulcognatha) blairi* (Bentanachs & Vives, 2009) comb. n.

#### Description.

Head and mandible enlarged in both sexes; gena acutely produced; clypeus strongly concave, very short; labrum reduced and not visible from dorsal view; mandible basally broadened, in male conspicuously, and distal half antero-laterally of mandible in both sexes with prominently developed concavity (Fig. 2A; 3D). Apex of mesosternal process concealed by metasternum; antenna and tibia with chromatic sexual dimorphism.

#### Etymology.

The new subgenus name is a combination of the Latin word sulcus (meaning, “furrow”) and the Greek word gnathus (meaning, “jaw”), and is a reference to the deep antero-lateral furrow in the mandible of both genders. The name is feminine.

#### Included species.

This subgenus includes so far only the type species.

### Hexamitodera (Sulcognatha) blairi

(Bentanachs & Vives, 2009) comb. n.

http://species-id.net/wiki/Hexamitodera_blairi

Fig. 1C, D

#### Redescription.

Male. Head abnormally developed (Fig. 1C; 2A), dorsally uniformly finely punctured, with a fine longitudinal furrow reaching the base. Temple straight, not protruded under the forehead in dorsal view, distance between temples narrower than anterior pronotal margin. Gena elongate and acutely produced exteriorly, anterolaterally rounded, maximal width distinctly wider than forehead and as wide as pronotum. Mandible as long as head, twice as long as scape, maximal width as long as scape, strongly curved inwards, forming a transverse ellipse in closed position; apex flattened, shovel-shaped, bidentate, internal tooth obtuse; molar margin with strong basal tooth. Antenna slightly surpassing elytral half, articles 1**–**2 spineless, glabrous, red-brown, 3**–**6 with small apical spines, fine short pubescence, testaceous to brass-colored, 7**–**11 spineless, sparsely pubescent, dark-grey/brown, 11 apically rounded. Prosternal process projecting over posterior border of procoxae. Elytra 2.5 times as long as prothorax and head without mandibles, with brass-colored velvety pubescence. Femur dark-brown to black, tibia brass-colored to testaceous.

Female. Mandible as long as scape, flattened, acutely pointed at the apex, between tooth of apex and base continuous, not strongly curved inwards, molar margin without tooth. Gena forming an obtuse angle, their maximal width narrower than width between eyes, nearly as wide as forehead. Antenna dark blue with metallic reflections, sparsely pubescent. Elytron 3.3 times as long as prothorax and head excluding mandibles, metallic blue.

#### Geographical distribution.

Sulawesi.

### Niisatochroma celebiana

Vives & Bentanachs, 2010

http://species-id.net/wiki/Niisatochroma_celebiana

#### Discussion.

In *Niisatochroma celebiana*, the sole species in this genus, the chromatic gender dimorphism (brass brownish tones in male, bluish-green in female), dorsal body pubescence, prominent developed elytral costae and short limbs (see Vives and Bentanachs 2010) indicate a close relationship with *Hexamitodera semivelutina* and *Hexamitodera (Sulcognatha) blairi*. The thickened antenna and distinct apical spines on the antennomeres (Vives and Bentanachs 2010) however suggest that this species does not belong to the same lineage as *Hexamitodera (Sulcognatha) blairi*. Like *Hexamitodera (Sulcognatha) blairi*, *Niisatochroma celebiana* might deserve subgeneric status within *Hexamitodera*, however, unfortunately no specimens of this rare species were available for a detailed study and therefore the generic status has to be retained for the moment.

#### Geographical distribution.

Sulawesi.

## Discussion

### Taxonomy

Contrary to the generic characters provided for *Parandrocephalus* (Bentanachs and Vives 2009), the mandible of *Hexamitodera (Sulcognatha) blairi* does not possess a dorsomedian carina (Fig. 2A; 3D) and the elytra have prominently (and not weakly) developed carina (Fig. 1 C, D). Consequently the generic position of *Hexamitodera (Sulcognatha) blairi* had to be reconsidered.

The separate treatment of sexual and non-sexual traits has revealed a suite of apomorphic characters (Table 1) that allows a more coherent interpretation of relationships of *Hexamitodera (Sulcognatha) blairi*. Actually, the chromatic gender dimorphism (brownish tones in male, blue in female), body pubescence, prominent developed elytral costae, short legs and antennae of *Hexamitodera (Sulcognatha) blairi* clearly belong to *Hexamitodera* (Table 1). The relationship becomes already evident in the comparison of the dorsal habitus of the females of *Hexamitodera (Sulcognatha) blairi* and *Hexamitodera semivelutina* (Fig. 1D, F).

**Table 1.** Generic differences of *Parandrocephalus* and *Hexamitodera*<br/>

**Character**	***Parandrocephalus***	***Hexamitodera***
Body chromatic sexual dimorphism	absent	present
Shape male mandibular	vertically flattened, with dorsomedian carina, interiorly concave, distal portion interiorly with deep concavity	horizontally flattened, dorsally straight, dorsomedian carina absent, dorso-lateral border, externally with shallow to deep concavity
Pubescence male head + pronotum	absent	distinct
Elytral costae + pubescence	weak	distinct
Light pubescence at female abdominal sternites	first to fourth	first
Metatibia distally flattened and broadened	distinct	moderate
Width of metatibia distally	~ metafemora	<metafemora

The lacking sexual dimorphism in mandible and head of *Hexamitodera semivelutina* suggests an ancestral relationship with *Hexamitodera (Sulcognatha) blairi*, while the enlargement and complex structure of the mandible in *Hexamitodera (Sulcognatha) blairi* indicate a more derived status. I hypothesise that the grades of differentiation in the mandible in the two *Hexamitodera* species represent the basal and terminal end of an evolutionary transformation series (Fig. 3A-D) of male adaptations to adjust on the female during the mating. The interior shape of the male mandible in *Hexamitodera (Sulcognatha) blairi* fits well with the posterior constriction of the female prothorax, while the antero-distal mandibular concavity is perfectly suited to accommodate the female profemora. The large mandibles and their adjustment on the female during the mating are evolutionarily advantageous because they facilitate a successful (possibly prolonged) fertilization and contribution to the gene pool. In this context, the inclusion of *Hexamitodera (Sulcognatha) blairi* into *Hexamitodera* provides an interesting hypothesis for testing evolutionary processes that select for large mandibles and co-adaptations.

It might be asked if the modifications in head and mandible of *Hexamitodera (Sulcognatha) blairi* justify the establishment of a new genus, however, particularly on the basis of morphology it is difficult to assess at which point a phylogenetic distance passes a subgeneric or generic boundary, and additionally there is no operational definition for such boundaries. Mayr (1969) pragmatically defined a genus as “a taxonomic category containing a single species, or a monophyletic group of species, which is separated from other taxa of the same rank (other genera) by a decided gap.”

To my knowledge, *Hexamitodera semivelutina* and *Hexamitodera (Sulcognatha) blairi* are from other taxa of the same rank unambiguously distinguished by apomorphic characters (Table 1) and the lack of intermediate mandible and head forms, indeed representing a certain gap, at the very most justifies the institution of a new subgenus.

### Convergence in secondary sexual characters

The extraordinarily developed mandibles in males of *Parandrocephalus* and *Hexamitodera (Sulcognatha) blairi*,evidently showing distinct features (Table 1; Fig. 2; 3D, F), should be interpreted as convergent adaptations, possibly for mate-guarding and the fitting of the male mandible to the posterior constriction of the female prothorax. While the mandible structure might contain useful taxonomical information, the enlargement alone is not a good indicator for relationships since it has also independently evolved in males of other Callichromatini genera (e.g. *Aphrodisium niisatoi* Vives & Bentanachs, 2007 and *Huedepohliana superba* (Aurivillius, 1910)), and also in phylogenetically more distant beetle taxa, such as Prioninae (Cerambycidae), Manticorini (Carabidae) and Lucanidae.

Heller (1916) considered the opened procoxal cavity in *Parandrocephalus* and the African Callichromatini genus *Dictator* Thomson, 1878 (as well having enlarged mandibles) as atypical for this tribe and indicator for a closer relationship, however, such combination is also observed in the phylogenetically distant groups already mentioned before and might represent a convergent co-adaptation. The opened procoxal cavities possibly allow a greater deflection of the procoxa and the strong bend of forebody and abdomen while the mandibles and prolegs keep close contact with the female prothorax during the mating. There might be indeed a Gondwanian relationship between African and Asian Callichromatini taxa, however, I think a detailed phylogenetic analysis based on a larger set of morphological or/and biochemical features is needed to present a robust hypothesis of actual relationships.

### Cerambycid-Geography and the Wallace line

Sundaland (including Borneo, Sumatra and Java) is assumed to be zoogeographically separated from ‘Wallacea’ (comprising Sulawesi and the Philippines except for Palawan) (Dickerson 1928) by the Wallace line (Huxley 1868), which is supported by distributional patterns e.g. in mammals and birds (Wallace 1859, 1860; Huxley 1868), cicadas (de Boer and Duffels, 1996), butterflies (see Vane-Wright and de Jong 2003 for a review) and hawkmoths (Beck et al. 2006).

The distribution of Cerambycidae taxa has been only sporadically treated in respect to the Wallace line and there is no statistical approach identifying geographical patterns.

Actually there are several closely related Cerambycidae taxa that occur on both sides of the Wallace line, e.g. the subspecies of *Xixuthrus microcerus* White, 1853 (Prioninae) (Komiya and Lorenc 2006), species of *Komiyandra* Santos-Silva, Heffern & Matsuda, 2010 (Parandrinae) (Santos-Silva et al. 2010) and *Chloridolum* Thomson, 1864 (Callichromatini) (Vives, in litt.), a pattern indicating more recent dispersal and colonization events.

The genera *Xystrocera* Blanchard, 1845 (Xystrocerini) (Martins 1960; Heffern 2005) and *Distenia* (Disteniinae) Lepeletier & Serville, 1828 (Gressit 1959; Heffern 2005; Santos-Silva and Hovore 2007) occur not only on both sides of the Wallace line, but also on other continents (Africa and South America), suggesting Gondwanian relationships and longer vicariance processes.

However, in both, the South-East Asian taxa that occur on both sides of the Wallace line and pantropical taxa, distributional patterns might also be influenced by transoceanic dispersal of larvae in drifting logs (see Holzapfel and Harrell 1968; Peck 1994).

The Wallace line holds for the tribe Tmesisternini (Lamiinae), which is highly diversified in New Guinea and Sulawesi but nearly absent in Sundaland (Heffern 2005), and the Callichromatini. Only two (*Chloridolum* Thomson, 1864, and *Pachyteria* Audinet-Serville, 1833) (Niisato 2001; Vives and Bentanachs 2010) of the 20 Callichromatini genera that are listed for Borneo (Heffern 2005) are found in Sulawesi. While some of the *Chloridolum* species that occur on both sides of the Wallace line are morphologically very similar (Vives, in litt.), other species reported for Sulawesi differ from con-generics in Sundaland and might belong to another genus (Vives and Bentanachs 2010).

There appear to be clear trends in some Cerambycidae tribes supporting the Wallace line, nevertheless, the examples predating such line call for a proper statistical analysis of morphological or biochemical characters to clarify phylogeographical relationships.

According to the current state of knowledge, the genus *Parandrocephalus* did not cross the Wallace line, supporting the idea that *Parandrocephalus* and *Hexamitodera* are indeed examples for convergent evolutionary processes within two zoogeographically distinct realms.

## Supplementary Material

XML Treatment for Parandrocephalus

XML Treatment for Parandrocephalus eversor

XML Treatment for Hexamitodera

XML Treatment for Hexamitodera semivelutina

XML Treatment for Sulcognatha

XML Treatment for Hexamitodera (Sulcognatha) blairi

XML Treatment for Niisatochroma celebiana
